# Perioperative fatigue in patients with diffuse glioma

**DOI:** 10.1007/s11060-020-03403-0

**Published:** 2020-01-23

**Authors:** Stine Schei, Ole Solheim, Asgeir Store Jakola, Lisa Millgård Sagberg

**Affiliations:** 1 Department of Public Health and Nursing, Norwegian University of Science and Technology, Trondheim, Norway; 2 Department of Neuromedicine and Movement Science, Norwegian University of Science and Technology, Trondheim, Norway; 3 Department of Neurosurgery, St. Olavs Hospital, Trondheim, Norway; 4 Department of Neurosurgery, Sahlgrenska University Hospital, Gothenburg, Sweden; 5 Institute of Neuroscience and Physiology, University of Gothenburg, Sahlgrenska Academy, Gothenburg, Sweden

**Keywords:** Brain neoplasms, Glioma, Fatigue, Surgery, Perioperative period

## Abstract

**Purpose:**

Few studies have assessed fatigue in relation to glioma surgery. The purpose of this study was to explore the prevalence of pre- and postoperative high fatigue, perioperative changes, and factors associated with pre- and postoperative high fatigue in patients undergoing primary surgery for diffuse glioma.

**Methods:**

A total of 112 adult patients were prospectively included. Patient-reported fatigue was assessed before and one month after surgery using the cancer-specific European Organization for Research and Treatment of Cancer questionnaire fatigue subscale. The scores were dichotomized as high fatigue (≥ 39) or low fatigue (< 39). A change in score of ≥ 10 was considered as a clinically significant change. Factors associated with pre- and postoperative high fatigue were explored in multivariable regression analyses.

**Results:**

High fatigue was reported by 45% of the patients preoperatively and by 42% of the patients postoperatively. Female gender and low Karnofsky Performance Status (KPS) were associated with preoperative high fatigue, while postoperative complications, low KPS and low-grade histopathology were associated with postoperative high fatigue. In total 35/92 (38%) patients reported a clinically significant improvement of fatigue scores after surgery, 36/92 (39%) patients reported a clinically significant worsening of fatigue scores after surgery, and 21/92 (23%) patients reported no clinically significant change in fatigue scores after surgery. Patients with low-grade gliomas more often reported low fatigue before surgery and high fatigue after surgery, while patients with high-grade gliomas more often reported high fatigue before surgery and low fatigue after surgery.

**Conclusions:**

Our findings indicate that fatigue is a common symptom in patients with diffuse glioma, both pre- and postoperatively. Perioperative changes were frequently seen. This is important knowledge when informing patients before and after surgery.

**Electronic supplementary material:**

The online version of this article (10.1007/s11060-020-03403-0) contains supplementary material, which is available to authorized users.

## Introduction

Diffuse gliomas are the most common types of primary brain tumors [1]. Due to their infiltrative growth pattern they cannot be cured [2], and the median survival is up to approximately 15 years for patients with low-grade glioma (LGG) [3] and 10–12 months for patients with high-grade glioma (HGG) [4, 5]. The incurable nature of diffuse gliomas thus makes preservation of quality of life a paramount factor to consider in treatment decision making. Even though extensive surgical resections may prolong survival in both LGG and HGG [6–8], there is a significant risk of adverse effects such as postoperative complications and acquired neurological deficits. However, intact neurological functions after surgery do not necessarily guarantee preservation of quality of life as more subtle and subjective symptoms may be undetected. In fact, cancer patients indicate fatigue as one of the most troublesome symptom related to cancer and its treatment [9], and the symptom is also common in glioma patients with an estimated prevalence of 36–82% [10–14].

Cancer-related fatigue is complex and can be influenced by treatment-related factors, and physical and emotional consequences of the diagnosis [15]. The underlying biological mechanisms of fatigue are poorly understood, but there is growing evidence that elevated levels of proinflammatory cytokines plays an important role, at least in extracranial cancers [16]. Proinflammatory cytokines are either released by immune cells following infection, by the tumor itself, or by tissue damage from surgery and/or adjuvant treatment [15]. In glioma patients, fatigue has mainly been studied in relation to oncological treatment [13, 17–20], and studies have found that fatigue is a prominent symptom already prior to oncological treatment [13, 20]. Fatigue is also found to have a negative impact on glioma patients’ health-related quality of life (HRQOL) [11, 14, 21], and to be a negative prognostic factor for survival in patients with HGG [19]. However, there is limited knowledge about fatigue in the perioperative neurosurgical setting, and there is a lack of longitudinal studies with fatigue as the primary outcome in this patient group.

We hypothesized that fatigue is a common symptom in the perioperative course and that the prevalence of fatigue may increase after surgery. The aims of this prospective study were therefore (1) to explore the prevalence of pre- and postoperative high fatigue in patients undergoing primary surgery for diffuse glioma, (2) to investigate perioperative changes, and (3) to explore patient- and treatment-related factors associated with pre- and postoperative high fatigue.

## Methods

### Study design and population

All patients aged ≥ 18 years that underwent primary surgical resection under general anesthesia for a grade II–IV glioma at the neurosurgical department at St. Olavs Hospital (Trondheim, Norway) from September 2011 through November 2015 were assessed for inclusion. This department serves a defined geographic catchment region with a population of approximately 720,000, ensuring a population-based referral. In total 112 patients were included in the study and filled out the European Organization for Cancer Treatment (EORTC) QLQ-C30 questionnaire at baseline. A flow chart of the inclusion process is presented in Supplementary Fig. 1. There was no significant difference in age (p = 0.756) nor Karnofsky Performance Status (KPS) (p = 0.095) between included patients and those without informed consent. Twenty patients (18%) were lost to follow up at 1 month, which left 92 patients with complete pre- and postoperative data. All tumors were histopathologically verified by a neuropathologist according to the 2007 World Health Organization-classification [22].

### The EORTC QLQ-C30 questionnaire

EORTC QLQ-C30 (version 3.0) is a validated and widely used questionnaire for HRQOL in cancer patients [23]. It contains 30 questions with five functioning domains, a global health status, six single-item scales, and three symptom scales. Fatigue is included as an unidimensional subscale and comprises three items assessing the physical domain of symptom intensity during the past week: “Did you need to rest?”, “Have you felt weak?”, and “Were you tired?”. Each question is answered on a four-point ordinal scale, where 1 is described as “not at all”, 2 as “a little”, 3 as “quite a bit”, and 4 as “very much”. The fatigue subscale has a high level of internal consistency, as determined with a Cronbach’s alpha of 0.88.

### Data collection and variables

The patients completed the Norwegian translated EORTC-questionnaire at admission 1–3 days before surgery. Follow-up assessments were performed by structured telephone interviews by a study nurse approximately 30 days postoperatively (median 31 days; range 23–63, mean 33 days ± 6.9). Assistance from proxies was used when the patients were too ill to answer, had considerable cognitive impairments or severe communication problems (5% of all follow-up interviews).

Demographic and clinical data were retrospectively obtained from electronic medical records (six regional hospitals and one university hospital). Preoperative symptoms were defined as new and/or increased tumor-related symptoms that was recorded in the medical journal prior to surgery. Only new or worsened language and/or motor deficits at discharge confirmed as persistent by patients at 30 days were included in the postoperative analyses. Charlson Comorbidity Index (CCI) was used to classify comorbidity [24], and complications during the first 30 postoperative days were graded as suggested by Landriel Ibañez et al. [25]. KPS was scored by the operating neurosurgeon just before surgery, while the postoperative scores were scored by a trained study nurse based on information from the telephone interviews. In one patient preoperative KPS was missing, and a retrospective estimation based on notes from the medical record was done to classify the patient as functionally dependent (KPS < 70) or functionally independent (KPS ≥ 70). Pre- and postoperative tumor volumes and tumor locations were obtained from pre- and postoperative magnetic resonance imaging (MRI) scans. The tumor volumes were estimated by a neurosurgeon by applying the volume formula V = 4π × r^3^/3, based on perpendicular tumor diameters. The volume of pathological contrast-enhancement and necrotic tissue within the contrast-enhancing borders were used in contrast-enhancing tumors, while the entire volume as seen in T2/FLAIR sequences was used in tumors without contrast-enhancement.

### Statistical analyses

In accordance with the EORTC scoring manual, the fatigue subscale were transformed to a 0–100 scale with higher scores indicating more severe fatigue [26]. To identify only patients with clinically significant and severe fatigue, the fatigue scores were further grouped as “high fatigue” (≥ 39) or “low fatigue” (< 39) as recommended by Giesinger et al. [27].

All statistical analyses were performed using SPSS version 25.0. Statistical significance was defined as p < 0.05. The correlation between fatigue and categorical factors was explored using Pearson´s χ^2^ tests. Fisher exact test was used when the expected number of cells was ≤ 5. Q–Q plots and Shapiro–Wilk tests were used to test for normal distribution for continuous variables. Means are presented if data was normally distributed, while medians are presented if data was skewed. Student’s sample *t* test or Mann–Whitney U tests were carried out to compare continuous variables depending on whether data were normally distributed or skewed. Binary logistic regression analyses were performed, and only univariables with a statistical trend (p < 0.1) were included in the multivariable models. The potential collinearity between variables was assessed with correlation coefficients, tolerance values, and the variance inflation factor (VIF). The Hosmer–Lemeshow goodness-of-fit test was used to determine goodness of fit of the logistic regression model, and the Nagelkerke R square value was used to assess how much variation in the dependent variable that could be explained by the model. Perioperative changes in fatigue were examined in a cross-table, and possible associated factors at group level. High fatigue before surgery and low fatigue after surgery was defined as a score of ≥ 39 preoperatively, and < 39 at postoperative follow-up. While low fatigue before surgery and high fatigue after surgery was defined as a score of < 39 preoperatively, and a score of ≥ 39 postoperatively. To assess clinically relevant change in fatigue score, the previously published minimal clinically important difference score of ± 10 for patients with brain cancer was applied [28].

### Missing data

In one patient, one fatigue-item was missing at baseline. This item was therefore imputed according to EORTC scoring manual by assuming that the missing item value was equal to the average of those other two items scored by the patient [26].

## Results

### Preoperative fatigue and possible associated factors

In Table 1, preoperative data for patients with high and low levels of fatigue symptoms are compared. As seen, 50/112 patients (45%) reported high fatigue the last week before surgery, and women reported high fatigue more frequently than men (66% vs. 34%, p = 0.001). Patients with functional dependency reported more high fatigue compared to those with functional independency (79% vs. 40%, p = 0.006). Also, preoperative high fatigue was more common in patients with nausea/vomiting (89% vs. 11%, p = 0.010), motor deficits (73% vs. 27%, p = 0.016), and dizziness/balance/coordination problems (66% vs. 34%, p = 0.009). In contrast, high fatigue was less common in patients with seizures (30% vs. 70%, p = 0.010).

**Table 1:** Baseline data, prevalence of preoperative fatigue, and possible associated factors, n = 112

Characteristics	High fatigue (N = 50) n/N (%)	Low fatigue (N = 62) n/N (%)	*p* value
Age (years), median (range)	56 (18–80)	62 (23–80)	0.482
Gender			**0.001**
Female	25/38 (66)	13/38 (34)	
Male	25/74 (34)	49/74 (66)	
Histopathology			0.721
Diffuse low-grade glioma	13/31 (42)	18/31 (58)	
High-grade glioma	37/81 (46)	44/81 (54)	
Location			
Frontal	15/43 (35)	28/43 (65)	0.101
Temporal	11/26 (42)	15/26 (58)	0.785
Parietal	3/3 (100)	0/3 (0)	0.086
Occipital	0/0 (0)	0/0 (0)	N/A
Cerebellum/brainstem	2/2 (100)	0/2 (0)	0.197
Basal ganglia^a^	1/5 (20)	4/5 (80)	0.378
Multiple lobes	18/33 (55)	15/33 (45)	0.173
Lateralization			
Right	26/55 (47)	29/55 (53)	0.582
Left	23/52 (44)	29/52 (56)	0.935
Bilateral/midline	1/5 (20)	4/5 (80)	0.378
Preoperative KPS^b^			**0.006**
≥ 70	39/98 (40)	59/98 (60)	
< 70	11/14 (79)	3/14 (21)	
Preoperative symptoms^c^			
Headache	23/41 (56)	18/41 (44)	0.064
Seizures	13/44 (30)	31/44 (70)	**0.010**
Cognitive change	19/36 (53)	17/36 (47)	0.233
Nausea/vomiting	8/9 (89)	1/9 (11)	**0.010**
Dizziness/balance/coordination problems	19/29 (66)	10/29 (34)	**0.009**
Visual disturbance	4/6 (67)	2/6 (33)	0.405
Language problems	13/28 (46)	15/28 (54)	0.826
Cranial nerve deficits	9/14 (64)	5/14 (36)	0.114
Motor deficits	11/15 (73)	4/15 (27)	**0.016**
CCI > 1^d^	2/5 (40)	3/5 (60)	1.0
Preoperative corticosteroids			0.327
Yes	32/66 (48)	34/66 (52)	
No	18/46 (39)	28/46 (61)	
Preoperative antiepileptic drugs			0.111
Yes	13/38 (34)	25/38 (66)	
No	37/74 (50)	37/74 (50)	
Preoperative tumor volume cm^3^, median (range)	25.22 (0.51–107.89)	20.39 (1.01–94.78)	0.303

Bold values indicate p < 0.05

^a^Basal ganglia/thalamus/corpus callosum/insula

^b^Karnofsky Performance Status score

^c^Some patients had multiple symptoms

^d^Charlson Comorbidity Index

Possible factors associated with preoperative high fatigue were further explored in a multivariable logistic regression analysis (Table 2). All preoperative factors in Table 1 were first tested as univariables. Of these, gender, KPS, and symptoms such as seizures, motor deficits, dizziness/balance/coordination problems and headache showed a statistical trend (p < 0.1) and were included in the multivariable model. There was no evidence of multicollinearity between the independent variables (correlation < 0.7, tolerance value > 0.1 and VIF < 10). One patient was an outlier and therefore excluded from the analyses and one patient missing exact KPS. As seen, female gender and low KPS were the only significantly associated factors for preoperative high fatigue in the multivariable model. Females had 3.3 times higher odds for preoperative high fatigue than men, and higher KPS reduced odds for high fatigue. The Hosmer–Lemeshow test was not significant (p = 0.085), implying that the regression model was a good fit. The model explained 30.4% of the variance in development of fatigue and correctly classified 76.4% cases.

**Table 2:** Possible associated factors for high fatigue at baseline (n = 110) and at postoperative follow-up (n = 92)

Variables in the binary regression model	Univariable analyses		Multivariable analyses	
	OR (95% CI)	*p* value	OR (95% CI)	*p* value
Possible associated factors for high fatigue at baseline, n = 110^a^				
Female	3.69 (1.61–8.44)	0.002	3.28 (1.29–8.31)	**0.012**
Preoperative KPS	0.94 (0.91–0.97)	0.001	0.95 (0.92–0.99)	**0.017**
Seizure	0.34 (0.15–0.76)	0.009	0.69 (0.25–1.91)	0.485
Motor deficits	4.01 (1.19–13.54)	0.025	1.96 (0.47–8.11)	0.350
Dizziness/balance/coordination problems	3.12 (1.28–7.58)	0.012	1.60 (0.56–4.56)	0.379
Headache	2.20 (1.00–4.85)	0.050	1.46 (0.57–3.72)	0.428
Possible associated factors for high fatigue at postoperative follow-up, n = 92				
Age	0.97 (0.94–0.99)	0.040	0.97 (0.93–1.01)	0.142
Low-grade glioma	2.95 (1.18–7.38)	0.021	4.20 (1.11–15.88)	**0.034**
Moderate and/or severe complications^b^	4.18 (1.40–16.55)	0.013	7.11 (1.65–30.55)	**0.008**
Postoperative KPS	0.94 (0.89–0.98)	0.006	0.91 (0.86–0.96)	**0.001**

Bold values indicate p < 0.05

*OR* odds ratio, *CI* confidence interval, *KPS* Karnofsky Performance Status score

^a^One patient excluded due to outlier and one patient missing exact KPS

^b^Landriel grade II–III

### Postoperative fatigue and possible associated factors

In Table 3, postoperative data for patients with high and low levels of fatigue symptoms are compared. As seen, 39/92 (42%) reported high fatigue one month after surgery, and patients with postoperative high fatigue were significantly younger than those with low fatigue (median age 54 years [range 20–76] vs. 62 years [range 18–80], p = 0.046). Patients with LGG more often reported high fatigue than those with HGG (61% vs. 34%, p = 0.019). Also, patients who experienced moderate and/or severe complications (Landriel grade II-III) more often reported postoperative high fatigue (73% vs. 27%, p = 0.008).

**Table 3:** Postoperative data, prevalence of postoperative fatigue, and possible associated factors, n = 92

Characteristics	High fatigue (N = 39), n/N (%)	Low fatigue (N = 53), n/N (%)	*p* value
Age (years), median (range)	54 (20–76)	62 (18–80)	**0.046**
Gender			0.304
Female	15/30 (50)	15/30 (50)	
Male	24/62 (39)	38/62 (61)	
Histopathology			**0.019**
Diffuse low-grade glioma	17/28 (61)	11/28 (39)	
High-grade glioma	22/64 (34)	42/64 (66)	
Location			
Frontal	14/36 (39)	22/36 (61)	0.586
Temporal	10/23 (44)	13/23 (56)	0.903
Parietal	2/2 (100)	0/2 (0)	0.177
Occipital	0/0 (0)	0/0 (0)	N/A
Cerebellum/brainstem	1/1 (100)	0/1 (0)	0.424
Basal ganglia	4/5 (80)	1/5 (20)	0.159
Multiple lobes	8/25 (32)	17/25 (68)	0.218
Lateralization			
Right	18/44 (59)	26/44 (41)	0.783
Left	21/45 (47)	24/45 (53)	0.417
Bilateral	0/3 (0)	3/3 (100)	0.259
Postoperative KPS^a^			0.969
≥ 70	33/78 (42)	45/78 (58)	
< 70	6/14 (43)	8/14 (57)	
Moderate and/or severe complications^b^	11/15 (73)	4/15 (27)	**0.008**
New neurological deficits^c^	6/13 (46)	7/13 (54)	0.767
CCI > 1^d^	2/4 (50)	2/4 (50)	1.0
Corticosteroids at follow up			0.367
Yes	7/13 (54)	6/13 (46)	
No	32/79 (40)	47/79(60)	
Antiepileptic drugs at follow up			0.768
Yes	15/37	22/37	
No	24/55	31/55	
Extent of resection (%), median (range)^e^	93.5 (31.8–100)	94.9 (24.0–100)	0.302
Adjuvant treatment < follow up			
Chemotherapy^f^	19/52 (36)	33/52 (64)	0.195
Radiotherapy	18/51 (35)	33/51(65)	0.124

Bold values indicate p < 0.05

^a^Karnofsky Performance Status score

^b^Landriel grade II–III

^c^Motor and/or language deficits at discharge confirmed as persistent by patients at 30 days

^d^Charlson Comorbidity Index

^e^N = 89 due to 3 missing MRI

^f^Only temozolomide (no patients had received procarbazine, lomustine and vincristine)

To identify factors possibly associated with postoperative high fatigue, all factors in Table 3 were first tested as univariables. Of these, age, histology, KPS and postoperative complications were further included in the multivariable model (p < 0.1) (Table 2). There was no evidence of multicollinearity between the independent variables. In the multivariable analyses, low-grade histopathology, low KPS and moderate and/or severe complications were statistically significantly associated with postoperative high fatigue. The multivariable regression model was a good model of fit (p = 0.372). The model explained 37.4% of the variance in fatigue and correctly classified 76.1% of cases.

### Change in fatigue and possible associated factors

In all 112 patients, the median preoperative fatigue score was 33.3 (range 0–100) and in the 92 patients with follow up data, the median postoperative EORTC fatigue score was 33.3 (range 0–100), p = 0.511. In total 35/92 (38%) patients reported a clinically significant improvement of fatigue scores after surgery, 36/92 (39%) patients reported a clinically significant worsening of fatigue scores after surgery, and 21/92 (23%) patients reported no clinically significant change in fatigue scores after surgery. Both patients with LGG and HGG had a median fatigue score of 33.3 before surgery, while patients with LGG had higher median fatigue scores after surgery (median = 44.4, 95% CI 30.1–54.0 vs. median = 33.3, 95% CI 29.9–41.6) (Supplementary Fig. 2).

Dichotomized dynamics of change in fatigue from baseline to 1 month after surgery are shown in Table 4. Of 92 patients, 15 (16%) reported low fatigue before surgery and high fatigue after surgery, 62 (68%) reported no change, and 15 (16%) reported high fatigue before surgery and low fatigue after surgery.

**Table 4:** Change in fatigue from baseline to 1 month postoperatively

	Postoperative, n (%)		
	High fatigue	Low fatigue	Total
Preoperative, n (%)			
High fatigue	24 (26)	15 (16)	39 (42)
Low fatigue	15 (16)	38 (42)	53 (58)
Total	39 (42)	53 (58)	92 (100)

Table 5 shows the frequency of fatigue change at group level and possible associated factors. As seen in this hypothesis-generating table, low fatigue before surgery and high fatigue after surgery was more common in patients with LGG compared to patients with HGG (25% vs. 12%). Also, patients with LGG less often reported high fatigue before surgery and low fatigue after surgery compared to patients with HGG (3% vs. 22%). Patients reporting perioperative change in fatigue seem to have larger preoperative tumor volumes compared to those without change. Of those with moderate and/or severe complications, 40% reported low fatigue before and high fatigue after surgery. Low fatigue both before and after surgery was more common among men (48% vs. 27%) and in patients with higher preoperative functional levels (KPS ≥ 70) (45% vs. 11%).

**Table 5:** Change in fatigue and possible associated factors, n = 92

Characteristics	Low fatigue both before and after surgery (N = 38) n/N (%)	High Fatigue both before and after surgery (N = 24) n/N (%)	High fatigue before and low fatigue after surgery^a^ (N = 15) n/N (%)	Low fatigue before and high fatigue after surgery^a^ (N = 15) n/N (%)
Age (years), median (range)	62 (34–79)	53 (20–76)	55 (18–80)	61 (23–74)
Gender				
Female	8/30 (27)	11/30 (37)	7/30 (23)	4/30 (13)
Male	30/62 (48)	13/62 (21)	8/62 (13)	11/62 (18)
Histopathology				
Diffuse low-grade glioma	10/28 (36)	10/28 (36)	1/28 (3)	7/28 (25)
High-grade glioma	28/64 (44)	14/64 (22)	14/64 (22)	8/64 (12)
Preoperative KPS^b^				
≥ 70	37/82 (45)	20/82 (24)	12/82 (15)	13/82 (16)
< 70	1/9 (11)	3/9 (33)	3/9 (33)	2/9 (22)
Postoperative KPS^b^				
≥ 70	34/78 (44)	21/78 (27)	11/78 (14)	12/78 (15)
< 70	4/14 (29)	3/14 (21)	4/14 (29)	3/14 (21)
Preoperative tumor volume cm^3^, median (range)	16.85 (1.91–86.19)	19.72 (0.51–103.26)	30.38 (1.50–107.89)	29.97 (1.01–94.78)
Extent of resection (%), median (range)	95.1 (36.1–100)	92.7 (45.4–100)	94.8 (24.0–100)	94.7 (31.8–100)
Moderate and/or severe complications^c^	4/15 (27)	5/15 (33)	0/15 (0)	6/15 (40)

^a^All of these patients had clinical important change

^b^Karnofsky Performance Status score

^c^Landriel grade II–III

## Discussion

This prospective study explored fatigue in relation to primary surgery in patients with diffuse gliomas. Our findings indicate that fatigue is a prominent symptom in this patient group, as almost half of the patients experienced high levels of fatigue both before and after surgery. Female gender and low KPS were factors associated with preoperative high fatigue, while moderate and/or severe complications, low-grade histology and low KPS were associated with high fatigue one month after surgery. At group level, just as many reported low fatigue preoperatively and high fatigue postoperatively, as high fatigue preoperatively and low fatigue postoperatively. Patients with LGG more often reported low fatigue before surgery and high fatigue after surgery, while patients with HGG more often reported high fatigue before surgery and low fatigue after surgery. Patients with large tumors more often reported perioperative change compared to patients with smaller tumors.

In the general Norwegian population, the median EORTC fatigue score is 28.8 and thereby lower than the pre- and postoperative median scores found in our glioma population [29]. In the single prior study with preoperative data that can be compared with ours the fatigue prevalence was almost twice as high [14]. The difference in patient selection, study design, assessment time point, and definition of fatigue may explain why the prevalence differed. The postoperative prevalence found in our study is comparable with a previous study of glioblastoma patients, where 48% reported fatigue at postsurgical baseline [13]. However, the lack of consensus in assessment of fatigue with respect to different questionnaires, cut-off scores and assessment time points hamper meaningful comparisons between studies. Also, most previous studies on fatigue in glioma patients are cross-sectional with strict inclusion criteria, where patients with KPS < 70 and cognitive impairments are often excluded [10–12, 14, 17, 18, 20, 30, 31].

We found female gender to be associated with high fatigue before, but not after surgery. As suggested by others, women may be more aware of, or more willing, to report their symptoms compared to men [32]. The finding may also be explained by that women, in general, experience stronger emotional reactions to illness than men [33]. Admittedly, our sample included twice as many men than women, which may have affected these results. The reported findings on the impact of gender in relation to fatigue varies in the literature. In glioma patients, Cheng et al. found no association between female gender and fatigue prior to surgery [14], while another study found female gender to be associated with fatigue in glioblastoma patients after surgery [13]. Further, a relationship between fatigue and female gender has been found both in patients with general cancer [34] and in the general population [29].

Poor functional status is a well-known negative prognostic factor for survival in patients with diffuse glioma [35, 36], and we found patients with low pre- and postoperative KPS also to have a slightly increased odds of high levels of fatigue. In accordance with our findings, a relationship between low KPS and preoperative high fatigue is found in a previous study as well [14], while another study found no relationship between KPS and fatigue at postsurgical baseline [13].

Low-grade histology was another independent factor for postoperative high fatigue, and patients with LGG had higher median postoperative fatigue scores, whereas the HGG group had stable median fatigue scores one month after surgery. This may seem surprising considering the poorer prognosis and often lower functional status in HGG patients [37]. Since most of the patients who were lost to follow up had HGG, selection bias may be an issue. Another explanation could perhaps be that patients with LGG are often younger and less symptomatic prior to surgery and may have higher expectations and obligations to carry on with the same activities, both at work and in their social life as before surgery, and thus experience a larger difference between their present and previous situation.

Of note, preoperative high fatigue appeared to be less common in patients with seizures, although not significant in the multivariable analyses. Seizures is a common symptom, especially in patients with LGG and in cases where the tumor is located in the frontal, temporal and parietal lobes [38]. Thus, this finding may perhaps have been confounded by the higher frequency of frontal tumors and low-grade histology in the “low fatigue” group. Preventing surgical complications are of always of importance and moderate and/or severe postoperative complications were found to be associated with postoperative fatigue. However, this finding was based on relatively few patients, and some of them also had several complications which makes further interpretation difficult.

Perioperative changes in fatigue were frequently seen on an individual level. Postoperative reduced mass effect or reduced peritumoral edema may explain why some patients experienced high levels of fatigue before surgery and low levels of fatigue after surgery. While inflammatory response due to tissue irritation/damage following surgery may explain why some experienced low fatigue before surgery and high fatigue after surgery. The tumor itself is also known to elicit inflammation. Patients with a perioperative change had seemingly larger preoperative tumor volumes. However, a previous cross-sectional study found no relationship between tumor size and fatigue in primary brain tumor patients [31], and no association between fatigue and extent of resection in glioblastoma patients at postsurgical baseline has been found [13].

The high pre- and postoperative prevalence and the perioperative change in fatigue may also be attributed to psychological and emotional responses to the cancer diagnosis and surgery. Some patients may be anxious and/or depressed after being diagnosed and treated for a life-threatening disease, while others may experience some relief after successful surgery.

To our knowledge, this is the first study to explore the implications of surgery on fatigue in patients with diffuse glioma, and the unselected study population increase the generalizability of our findings. The fatigue assessment was prospective and standardized using a validated cancer-specific questionnaire. Admittedly, when measuring fatigue as a defined end-point, it may seem more reasonable to use a fatigue-specific questionnaire that cover more than physical dimensions of fatigue [39]. However, challenges regarding data collection and high drop-out rates are known problems in longitudinal studies of glioma patients [40], and complicated forms may introduce selection bias. Thus, since the present study was part of a larger project already using the EORTC QLQ-C30 questionnaire, the fatigue subscale was used to ensure compliance and reduce the burden on patients. Other limitations that should be taken into account when interpreting the results are that our sample included almost twice as many men as women, and that psychosocial factors were not included in the analyses.

There is no ideal time point for assessing fatigue after surgery. The symptom tends to fluctuate over the course of the disease, and inappropriate timing of assessment can therefore result in failure to capture the true implications of surgery [41]. In the early postoperative period, it is more likely that fatigue can be affected by potential reversing contributory factors, such as analgesic, postoperative pain and transient postoperative complications. However, the symptom may also be a side effect of early initiated adjuvant treatment [42, 43]. The clinical experience is that many patients operated for intracranial tumors report fatigue that gradually weans over several months. Thus, a later postoperative assessments than at one month may seem more appropriate. However, in rapid progressive diseases like HGG too late assessments may reflect disease progression more than treatment, and in patients with stable disease too late assessments may be affected by response shifts as patients adapt to their new situation over time [44].

Knowledge about fatigue in the surgical setting may raise awareness among clinicians that fatigue is a prominent symptom in the perioperative setting. This may be important knowledge when informing patients about what to expect after surgery. Patients with primary brain tumors have expressed a need for more preparatory information about fatigue [21], and described uncertainty about symptoms and how they could cope with the changes [45]. In addition, our findings may provide foundation for further research. For example, the symptom is not much explored in LGG patients after undergoing repeated resections and adjuvant interventions [3].

## Conclusions

In this prospective study, we found fatigue to be a common symptom in patients with primary diffuse glioma, both before and after surgery. Female gender and low KPS were associated with high preoperative fatigue, and postoperative moderate and/or severe complications, low KPS and low-grade histopathology were associated with more postoperative fatigue. Perioperative change in fatigue was frequently seen. Since fatigue is likely to affect quality of life in glioma patients, knowledge of the symptom in the perioperative course is important when informing patients before and after surgery.

## Electronic supplementary material

Below is the link to the electronic supplementary material.

Supplementary file1 (PDF 300 kb)

Supplementary file2 (PDF 168 kb)

## Data Availability

The datasets generated during and/or analysed during the current study are not publicly available due to privacy concerns but are available from the corresponding author on reasonable request.
