# Supplementary material for: Perioperative fatigue in patients with diffuse glioma
Source: J Neurooncol. 2020 Jan 23;147(1):97–107. doi: 10.1007/s11060-020-03403-0 (PMC7075831; doi:10.1007/s11060-020-03403-0)
Supplement: Supplementary file 1 — Supplementary file1 (PDF 300 kb) [file 11060_2020_3403_MOESM1_ESM.pdf]

Journal of neuro-oncology

Perioperative fatigue in patients with diffuse glioma

Stine Schei, MSc, [1] **Ole Solheim, MD, PhD,** [2-3] **Asgeir Store Jakola, MD, PhD,** [2,4,5] **and Lisa Millgård Sagberg, PhD,** [1,3]

1 Department of Public Health and Nursing, Norwegian University of Science and Technology, Trondheim, Norway
2 Department of Neuromedicine and Movement Science, Norwegian University of Science and Technology, Trondheim, Norway
3 Department of Neurosurgery, St. Olavs Hospital, Trondheim, Norway
4 Department of Neurosurgery, Sahlgrenska University Hospital, Gothenburg, Sweden
5 Institute of Neuroscience and Physiology, University of Gothenburg, Sahlgrenska Academy, Gothenburg, Sweden

Corresponding Author´s name and current institution: Stine Schei, Department of Public Health and Nursing, Norwegian University of Science and Technology, Mauritz Hansens gate 2, 7030 Trondheim
Corresponding Author´s Email: stine.schei@ntnu.no

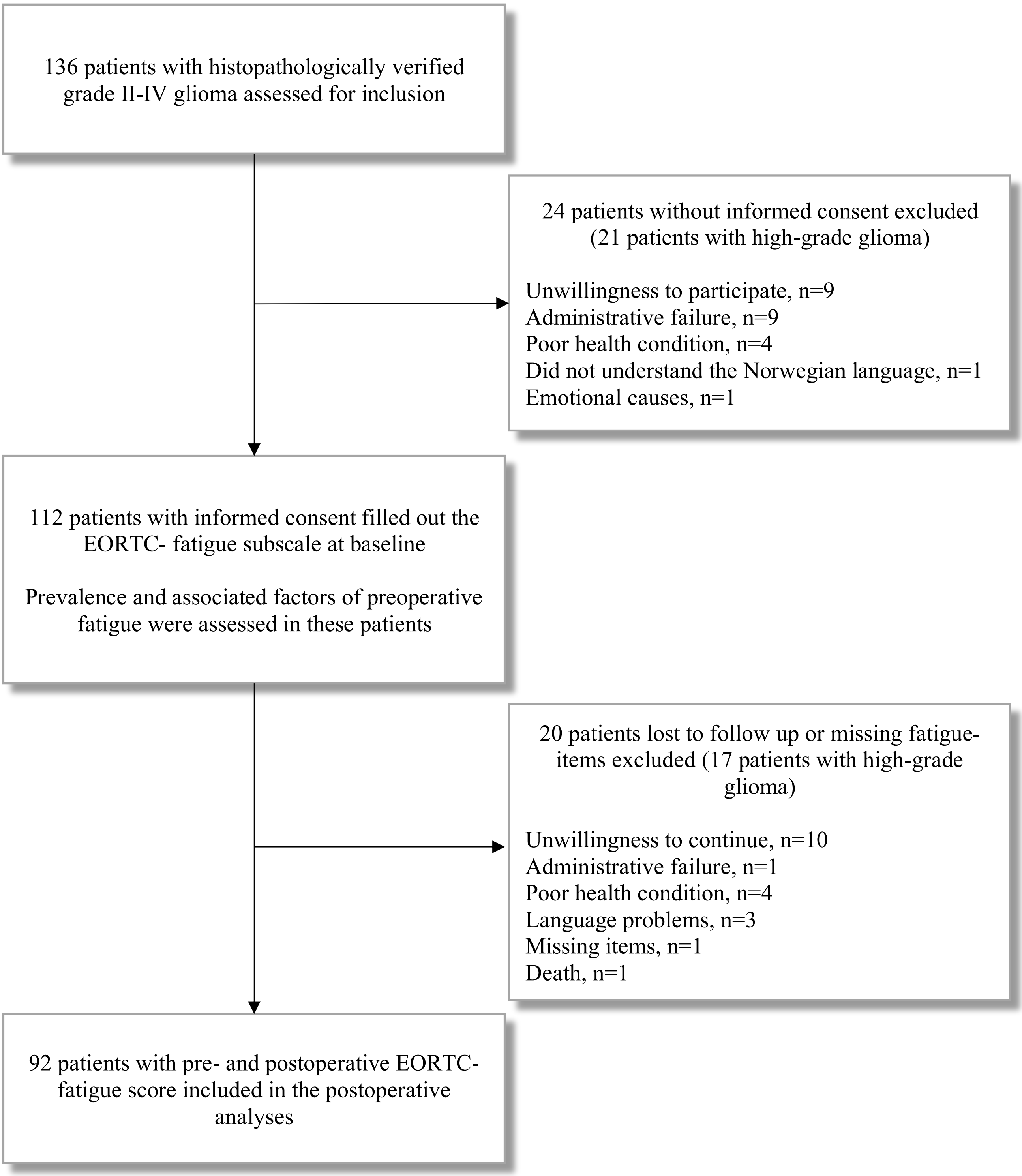

Fig. 1 Flowchart showing the inclusion process
